# The Illegitimate Child

**Published:** 1921-04-30

**Authors:** 


### The Illegitimate Child.

The National Council for the Unmarried Mother and her Child has appealed for our support of the provisions set forth in Captain Bowyer's Bastardy Bill, down for the second reading in the House of Commons yesterday. The Bill, which includes only three of the clauses of the measure introduced last year by Mr. Neville Chamberlain, has the support of many national societies, and resolutions in its favour have been passed by a number of local authorities and other public bodies. Its proposals have been a good deal watered down from those of the original Bill (in the hope of making it non-contentious), but it certainly goes far enough to give grounds for believing that it might be the means of saving many infant lives and doing something to alleviate the injustices under which the child of unmarried parents at present exists.

Mrs. H. A. L. Fisher (wife of the President of the Board of Education), who has always been a warm advocate of a Bill of this kind, brings out its two important points. In the first place, it makes good a serious legislative omission by enabling the parents of illegitimate children to legitimatise them by subsequent marriage, and in the second place it makes some effort to improve the provision of the urgently necessary intermediary between the father and mother of the illegitimate child — some authorised and properly qualified person, part of whose business it is to see that the mother of her illegitimate child receives an adequate sum for its maintenance from its father. The name Collecting Officer, Mrs. Fisher says, proved "unexpectedly alarming" to the average M.P., but presumably the discussions in Committee of Mr. Chamberlain's Bill made the utility of the official more obvious, and his nature more innocuous to man, for he emerged triumphant when much else was lost. The affiliation laws at present in operation do not sufficiently recognise the fundamental fact that "illegitimate children owe their existence, like other children, to both their parents, not to one only," and that both must share the responsibility for their upbringing. We agree that the plan of leaving practically the whole charge of the illegitimate child upon the mother has not succeeded in checking illegitimacy. The number of illegitimate births, after remaining stationary for a number of years, has lately increased, and of much greater importance is the suffering entailed on these children by the absence of paternal responsibility. The death-rate among illegitimate children is about double that of the legitimate, and it is likely to remain in that proportion as long as the average unmarried mother is obliged to nurse and rear her child at the same time that she must earn a living for it as well as for herself, because the contribution from the father is either hopelessly inadequate or is not maintained at all. We do not for a moment suggest that legislation, certainly not this modest little measure, would solve the problem of illegitimacy or of sex morality, but it would in all probability increase the father's sense of responsibility, and it would give many unfortunate children a better chance of survival and minister to the self-respect of others in after-life.

